# The Expression Levels and Cellular Localization of Pigment Epithelium Derived Factor (PEDF) in Mouse Testis: Its Possible Involvement in the Differentiation of Spermatogonial Cells

**DOI:** 10.3390/ijms22031147

**Published:** 2021-01-24

**Authors:** Noy Bagdadi, Alaa Sawaied, Ali AbuMadighem, Eitan Lunenfeld, Mahmoud Huleihel

**Affiliations:** 1 The Shraga Segal Department of Microbiology, Immunology, and Genetics, Faculty of Health Sciences, Ben-Gurion University of the Negev, Beer Sheva 8410501, Israel; bagdadin@post.bgu.ac.il (N.B.); alaasa@post.bgu.ac.il (A.S.); abumadig@post.bgu.ac.il (A.A.); 2 The Center of Advanced Research and Education in Reproduction (CARER), Faculty of Health Sciences, Ben-Gurion University of the Negev, Beer Sheva 8410501, Israel; lunenfld@bgu.ac.il; 3 Department of OB/GYN, Soroka Medical Center, Beer Sheva 8410501, Israel

**Keywords:** testis, spermatogenesis, in vitro differentiation of spermatogonial cells, pigment epithelium derived factor (PEDF), somatic cells, male fertility, 3D in vitro culture system

## Abstract

Pigment epithelium derived factor (PEDF) is a multifunctional secretory soluble glycoprotein that belongs to the serine protease inhibitor (serpin) family. It was reported to have neurotrophic, anti-angiogenic and anti-tumorigenic activity. Recently, PEDF was found in testicular peritubular cells and it was assumed to be involved in the avascular nature of seminiferous tubules. The aim of this study was to determine the cellular origin, expression levels and target cells of PEDF in testicular tissue of immature and adult mice under physiological conditions, and to explore its possible role in the process of spermatogenesis in vitro. Using immunofluorescence staining, we showed that PEDF was localized in spermatogenic cells at different stages of development as well as in the somatic cells of the testis. Its protein levels in testicular homogenates and Sertoli cells supernatant showed a significant decrease with age. PEDF receptor (PEDF-R) was localized within the seminiferous tubule cells and in the interstitial cells compartment. Its RNA expression levels showed an increase with age until 8 weeks followed by a decrease. RNA levels of PEDF-R showed the opposite trend of the protein. Addition of PEDF to cultures of isolated cells from the seminiferous tubules did not changed their proliferation rate, however, a significant increase was observed in number of meiotic/post meiotic cells at 1000 ng/mL of PEDF; indicating an in vitro differentiation effect. This study may suggest a role for PEDF in the process of spermatogenesis.

## 1. Introduction

Pigment epithelium derived factor (PEDF) is a glycoprotein that belongs to the serine protease inhibitor proteins superfamily but lost its protease inhibitory properties [1]. It is widely expressed in most body tissues [1,2] but the highest expression has been observed in the eye, liver, adult testis, ovaries, placenta and the pancreas [3]. In senescent cells, there is a significant decrease in its expression [4,5,6,7].

PEDF is a multifunctional protein with properties of neurotrophic, neuroprotective, gliastatic, anti-inflammatory, anti-tumorigenic, anti-angiogenic and antivasopermeability [8,9,10].

Several receptors have been suggested to participate in the PEDF mechanism of action: patatin-like phospholipase domain containing 2 (PNPLA2) [11,12], laminin receptor (LR) [13], lipoprotein receptor-related protein 5 (LPR5; Wnt co-receptor) [14] and cell surface F1F0-ATP synthase [15]. PEDF binds with high affinity to PNPLA2; therefore, it is regarded as the main receptor for PEDF [16,17].

Recently, a study conducted on human testes revealed PEDF protein in testicular tissue and particularly in testicular peritubular cells and extracellular matrix. Those finding suggested that the avascularity of the seminiferous tubules of the testis is likely due to the secretion of PEDF by Peritubular cells [17].

## 2. Results

### 2.1. Immunostaining of PEDF and Its Levels in Testicular Tissue and Sertoli Cells

**Figure 1:**
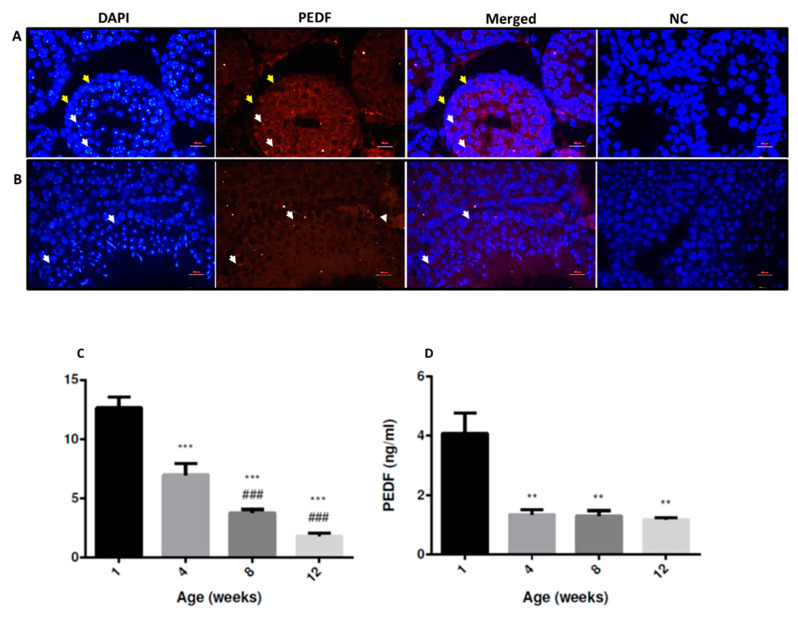
Immunostaining of pigment epithelium derived factor (PEDF) and its levels in testicular tissue and Sertoli cells. Paraffin embedded testicular tissue from 2-week-old (immature) mice (**A**) and from 8-week-old (mature) mice (**B**) were sliced, fixed on slides and immunofluorescence stained for PEDF (red) and DAPI (blue). Negative control (NC)—IF staining without the presence of primary antibody. PEDF levels were examined in testicular homogenates (**C**) and in Sertoli cells supernatants (**D**) by using specific ELISA. (**C**) N = 7–10. N—number of mice used for testis homogenates repeat. (**D**) N = 5–6. N—Number of experiments repeated for Sertoli cells supernatant. White arrows indicate stained cells, while yellow arrows indicate non-stained cells in the seminiferous tubule (**A**). ∗—Statistical significance of mice in comparison to 1w, #—Statistical significance of mice in comparison to previous age. ∗∗—*p* < 0.01, ∗∗∗—*p* < 0.001. ###—*p* < 0.001. Scale bar: 100 μm.

Our immunofluorescence (IF) staining results show the presence of PEDF in cells of the interstitial compartment and in most of the cells within seminiferous tubules of immature (2-week-old) (positive-stained cells in the seminiferous tubule were indicated by white arrows, while those did not stain were indicated by yellow arrows) and adult (8-week-old) mice (Figure 1A,B, respectively). PEDF levels were measured in the homogenates of testicular tissue from mice at different ages (1–12 weeks) (Figure 1C), and showed a significant decrease with age (in all examined ages) compared to 1 week-old, and in comparison to previous age (Figure 1C). PEDF levels were also measured in conditioned media of Sertoli cells from mice at different ages (1–12 weeks) (Figure 1D) and showed a significant decrease with age (in all examined ages) compared to 1 week (Figure 1D).

### 2.2. Cellular Localization of PEDF in Testicular Cells

**Figure 2:**
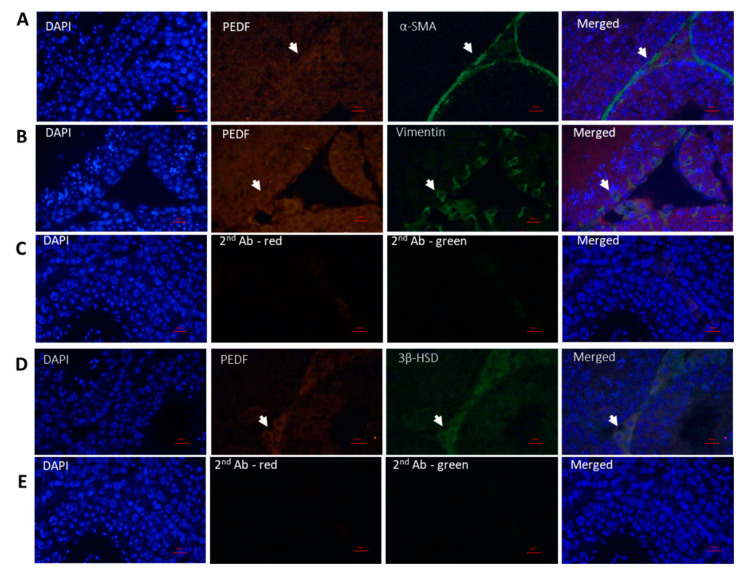

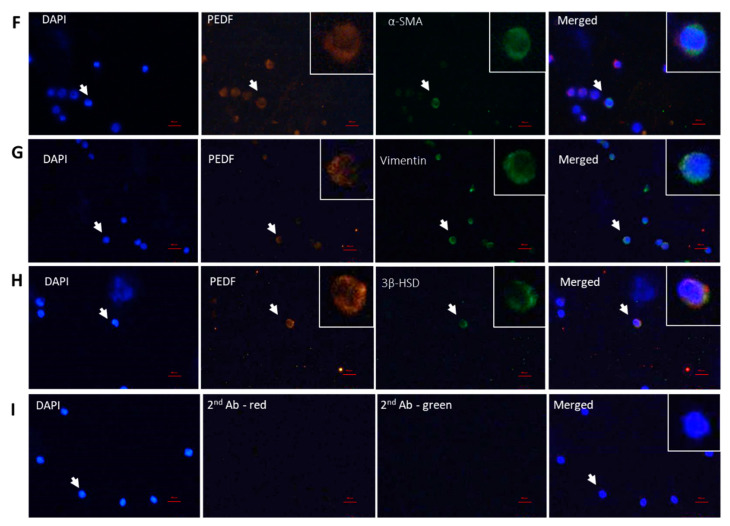
Cellular localization of PEDF in testicular somatic cells. Paraffin embedded testicular tissue from 6-week-old mice were sliced, fixed on slides and immunofluorescence double stained for DAPI (blue), PEDF (red) and for testicular somatic cells markers (green): (**A**) α-SMA (peritubular cells marker), (**B**) vimentin (Sertoli cells marker), (**C**) tubular NC—without primary antibody, (**D**) 3β-HSD (Leydig cells marker), (**E**) interstitial tissue NC—IF staining without the presence of primary antibody. In addition, methanol-fixed isolated testicular cells were immunofluorescent double stained for: (**F**) α-SMA, (**G**) Vimentin and (**H**) 3β-HSD. (**I**) NC—IF staining without the presence of primary antibody. Scale bar: 100 μm.

**Figure 3:**
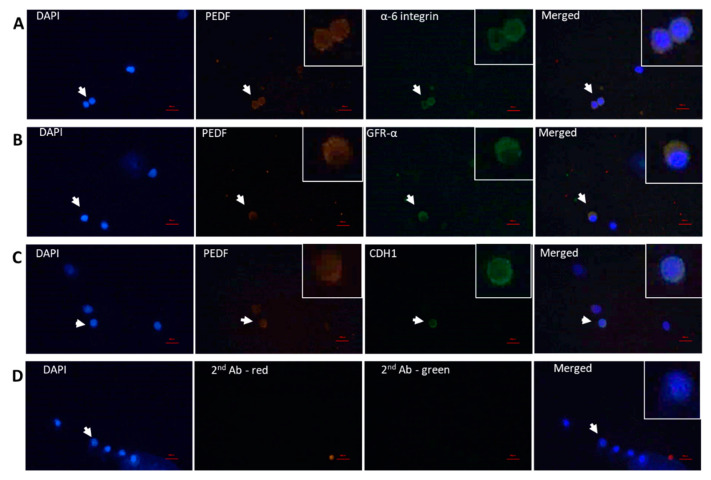

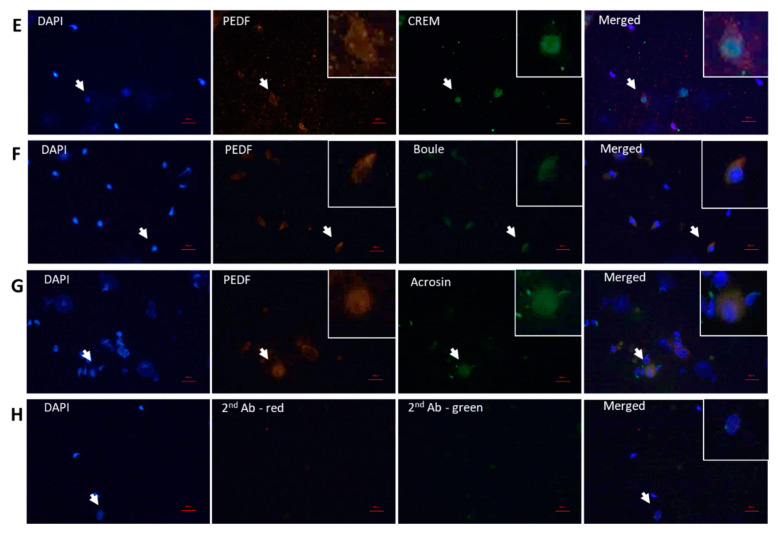
Cellular localization of PEDF in testicular spermatogenic cells. Methanol-fixed cells were used to stain premeiotic and meiotic/postmeiotic cells as described in Figure 2. (**A**) α6-integrin, (**B**) GFR-α, (**C**) CDH1, (**D**) and NC—IF staining without the presence of primary antibody. (**E**) CREM, (**F**) Boule, (**G**) Acrosin and (**H**) NC—IF staining without the presence of primary antibody. Scale bar: 100 μm.

In order to identify the cells that produce PEDF in the testicular tissue, testicular tissue of adult mice, and cells isolated from interstitial compartment and from seminiferous tubules of adult mice (8-week-old) were IF double stained for PEDF and markers specific for testicular somatic cells (α-SMA—peritubular cells, vimentin—Sertoli cells, 3βHSD—Leydig cells) (Figure 2) and spermatogenic cell markers (premeiotic -α-6-integrin, GFR-α, CDH1, meiotic—CREM and BOULE, postmeiotic—ACROSIN) (Figure 3). Our IF double staining of testicular tissues and cells isolated from seminiferous tubules or interstitial compartment show that PEDF is present in peritubular cells (Figure 2A,E), Sertoli cells (Figure 2B,F) and Leydig cells (Figure 2C,G). In addition, PEDF was present in the premeiotic cells (Figure 3A–C), meiotic cells (Figure 3D,E) and postmeiotic cells (Figure 3F).

### 2.3. Cellular Localization and Expression Levels of PEDF-R in Testicular Tissue and Cells

**Figure 4:**
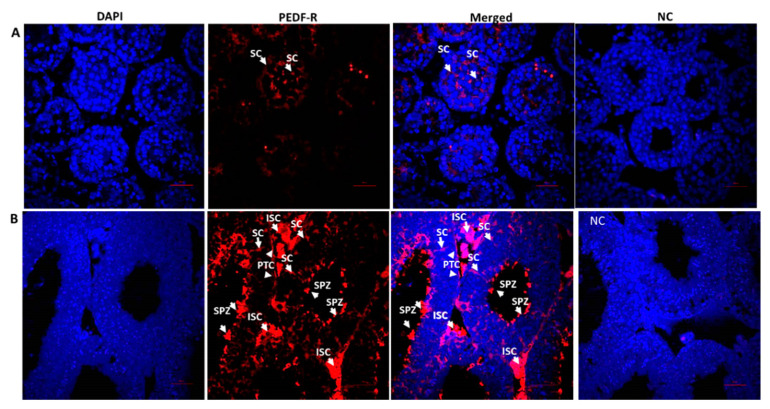

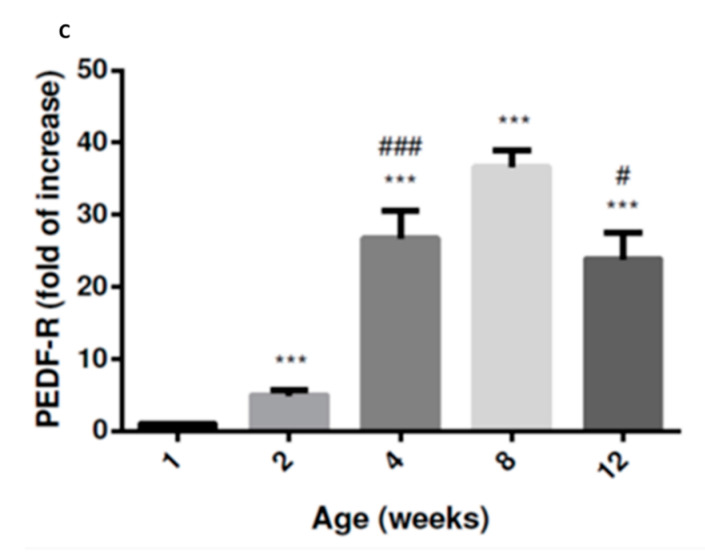
Cellular localization and expression levels of PEDF receptor (PEDF-R) in testicular tissue and cells. Paraffin embedded testicular tissue from 2-week-old mice (**A**) and 4-week-old mice (**B**) were used as described in Figure 1. Fixed sections were immunofluorescence stained for PEDF-R with specific antibodies (red) and DAPI (blue). NC were stained in the absence of the primary antibody. The expression levels of PEDF-R in testicular homogenates were evaluated by qPCR with specific primers (**C**). ∗—Statistical significance of mice in comparison to 1w. #—Statistical significance of mice in comparison to previous age. N = 4–6. N—number of mice used for testis homogenates repeat. ∗∗∗—*p* < 0.001. #—*p* < 0.05, ###—*p* < 0.001. Scale bar: 100 μm.

Our IF staining results showed the presence of PEDF-R in different cell types of the testis of immature (2-week-old) and mature (8-week-old) mice (Figure 4A and B, respectively). Staining of PEDF-R was pronounced in cells of the interstitial tissue (IST) and seminiferous tubules of testicular mature mice including Sertoli cells (SC), peritubular cells (PTC) and spermatozoa (SPZ) (Figure 4B). It seems that PDF-R is present in the branches of Sertoli cells in both immature and adult mice (clearer in the adult) (Figure 4A,B) and also in spermatozoa (SPZ) before release to the lumen of the seminiferous tubules of adult mice (Figure 4B).

The expression levels of PEDF-R in testicular homogenates of mice at different ages were examined using qPCR analysis (Figure 4C). A gradual and significant increase in the expression levels of PEDF-R was examined in testicular homogenates of mice from 1–4 weeks old. The expression levels of PEDF-R in testicular homogenates were significantly higher in all examined ages compared to 1 week. In addition, homogenates from 12-week-old mice showed a significant decrease in PEDF-R expression compared to 8-week-old mice.

### 2.4. Effect of PEDF on the Development of Spermatogenesis In Vitro

**Figure 5:**
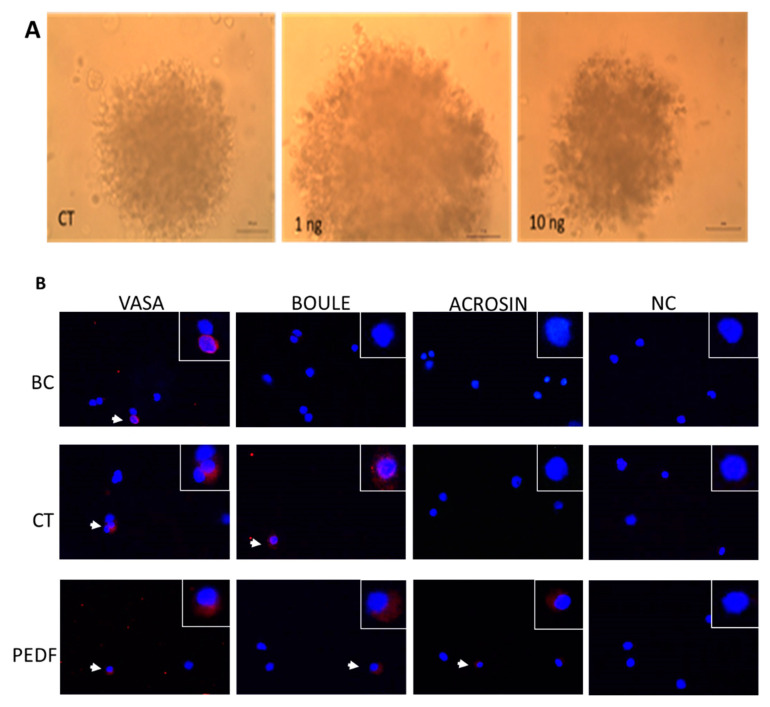

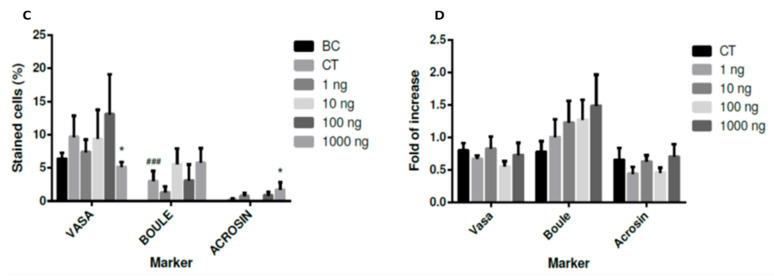
Effect of PEDF on the development of spermatogenesis in vitro. Cells isolated from seminiferous tubules of immature mice were cultured in methylcellulose in vitro culture system (MCS) as described in materials and methods. The cultures grew in the absence (CT) or in the presence of various concentrations of PEDF (1–10 ng/mL). The cultures were photographed after 4–5 weeks. ×20 magnification (**A**). The developed cultures in the absence of PEDF (CT) or presence of PEDF (PEDF) were collected, fixed and immunofluorescence stained in comparison to cells fixed before culture (BC) for spermatogenic markers: VASA (a premeiotic marker), BOULE (a meiotic marker) and ACROSIN (a meiotic/postmeiotic marker) (**B**). Negative control (NC)—immunofluorescence staining in the absence of the primary antibody. The percentage of VASA, BOULE and ACOSIN in the developed cultures were evaluated (**C**) and the expression levels of these markers were examined by qPCR analysis (**D**). #—Statistical significance in comparison to BC. ∗—Statistical significance in comparison to CT. (**B**) N = 4–5, *n* = 10. (**C**) N = 3–5, *n* = 10. N—number of experiments. Number of mice in each experiment ∗—*p* < 0.05, ###—*p* < 0.001. Scale bar: 100 μm.

In order to examine the role of PEDF in the process of spermatogenesis, cells from testicular seminiferous tubules of immature mice (7-day-old) were enzymatically isolated and cultured in MCS (as a three-dimension (3D) culture system) in the presence of StemPro medium, 10% KSR and the growth factors EGF, FGF, LIF and GDNF. Different concentrations of PEDF (1–1000 ng/mL) were also added to the cultures in order to determine the optimal concentration of PEDF that may be involved in the proliferation and/or differentiation of spermatogenic cells. After 4–5 weeks of culture, cells/colonies developed in vitro were documented by photographs (Figure 5A), and were collected to evaluate the presence of cells of different stages of spermatogenesis by IF staining (Figure 5B). The effect of PEDF on the percentages of developed cells from the different stages was quantified compared to control (CT) and to before culture (BC) (Figure 5C) and the expression levels of the markers for cells of these stages were also quantified by qPCR analysis (Figure 5D). Our results show development of colonies with different sizes both in control and following PEDF treatment (Figure 5). We could not identify significant differences in the in the number or size of the developed colonies between the control (CT) and following PEDF treatment (Figure 5A).

Cells and colonies developed in MCS in the presence or absence of PEDF were collected after 4–5 weeks of culture and stained for markers of cells from the different stages of spermatogenesis. Figure 5B represents staining of VASA (pre-meiotic), BOULE (meiotic) and ACROSIN (meiotic/post meiotic) from cells before culture (BC; to identify type of spermatogenic cells before culture) and from developed cells in MCS in the absence or the presence of PEDF in different concentration (1–1000 ng/mL). Our results show the presence of VASA but not BOULE or ACROSIN before culture (BC). However, cultures for 4–5 weeks in the absence (CT) or presence of PEDF (PEDF) showed staining for VASA, BOULE and ACROAIN.

Staining quantification results showed no effect of PEDF on the percentages of the VASA-positive stained cells (Figure 5C). However, at 1000 ng/mL, PEDF significantly decreased the percent of VASA-positively stained cells compared to CT (Figure 5C). The percentage of BOULE-positive cells was significantly increased in vitro (in the absence of PEDF; CT) compared to before culture (Figure 5C). However, addition of PEDF did not significantly affected the percentages of these cells (Figure 5C). On the other hand, even though ACROSIN positive cells were developed in vitro (CT) compared to before culture (BC) (Figure 5C), addition of PEDF significantly increased the percentage of ACROSIN-positive stained cells compared to CT (Figure 5C). In contrast, addition of PEDF in vitro, did not significantly affect the RNA expression levels of VASA, BOULE and ACROSIN compared to CT (Figure 5D).

## 3. Discussion

Our results show for the first time the localization of PEDF in mouse spermatogenic cells at different stages of development (premeiotic and meiotic/postmeiotic stages) as well as in the testicular somatic cells: Sertoli and Leydig cells. We also showed, as previously described by Windschüttl et al., 2015 [17] in human testes, that mouse peritubular cells produce PEDF. Our results also showed the presence of PEDF receptor in tubular cells (possibly the branches of Sertoli cells) and in cells of the interstitial compartment. These results may suggest possible autocrine and/or paracrine effects of PEDF in the regulation of testicular physiology (such as testicular development and development of spermatogenesis). It was shown that PEDF is a secreted protein which could be found intracellularly and in the extracellular compartment [3,4,12].

Our results also show the generation and expression of high levels of PEDF in testes of immature mice, and a significant decrease was examined with age (during sexual maturation). These results may indicate possible involvement of hormones (gonadotropins and testosterone) in the regulation of PEDF production and expression. In addition, our results show that Sertoli cell cultures produce PEDF; and the levels of PEDF were high in Sertoli cell cultures from immature mice and were significantly decreased in Sertoli cell cultures from adult mice. Again, it is possible to suggest that PEDF production by Sertoli cells could be under regulation of FSH and/or testosterone. Our results are in contrast to the group of Conte et al., 2018, who did not find expression and staining of PEDF in rat testicular cells [18]. This contrast could be related to the animals (rats) that were used by this group or antibodies and protocols they used, since we confirmed our results by different methods such as IF staining, ELISA (protein) and qPCR analysis. In addition, our results are in harmony with the group of Windschüttl et al., 2015, who demonstrated the presence of PEDF in human peritubular cells [17].

Our results showed the presence of PEDF-R within the seminiferous tubules cells of immature and mature mice as well as in the interstitial tissue of mature mice. PEDF-R protein staining in mature mice was stronger than protein staining in immature mice (Figure 4A,B). Compatible with this observation, RNA expression results of PEDF-R showed a significant increase in PEDF-R with age (Figure 4C). The presence of PEDF-R in testicular tissue was previously reported only in peritubular cells of the testis [17]. These observations may suggest a regulatory function for PEDF in the testis during immature and mature ages.

An opposite correlation was observed between protein levels of PEDF and PEDF-R (PNPLA2/ATGL). This may suggest a balance of activity of PEDF in immature and adult ages. This opposite correlation may indicate the possible distinct involvement of hormonal regulation of PEDF and PEDF-R that results in balanced activity of PEDF at the different ages. As mentioned before, PEDF is a multifunctional protein that was associated with several receptors in its mechanism of action. Opposite correlation between PEDF and the specific examined receptor PNPLA2/ATGL may indicate a restriction of PEDF activity in the specific mechanism exerted via this receptor in immature mice (high levels of PEDF, low levels of PEDF-R) and possible additional actions via other receptors, and vice versa in mature mice.

PEDF-R was first identified as adipose triglyceride lipase (ATGL), also known as patatin-like phospholipase domain-containing protein 2 (PNPLA2), and is considered as the main receptor for PEDF since it binds PEDF with high affinity. Therefore, in this work we used PNPLA2 as PEDF-R.

ATGL is an intracellular lipase that catalyzes the first step in lipolytic breakdown of triglycerides in lipid droplets stored in cells cytoplasm of adipose and non-adipose tissues. Eukaryotes deposit fatty acids as lipid droplets in all cells of the body for times of nutrient deprivation. These droplets are composed of triacylglycerol (TG) among other lipid molecules and there are numerous proteins that are associated with these lipid droplets [19,20].

In 2006, Notari et al. identified ATGL as a membrane associated protein that can exhibits phospholipase activity when it binds PEDF [11]. PEDF was also shown to exert its activity on ATGL on lipid droplets within the cells [12]. These data may suggest possible involvement of PEDF and its receptor in spermatogenesis process by modulation of metabolic activity of testicular cells.

Our results show that addition of PEDF to isolated cells from seminiferous tubules of immature mice (7-day-old) cultured in MCS induced the development of colonies, which were morphologically similar to control (absence of PEDF; CT) (Figure 5A). The developed cultures contained premeiotic, meiotic and postmeiotic cells (Figure 5B). Our results did not show a significant effect of PEDF on the percentages and expression levels of VASA (a premeiotic marker) and BOULE cells (a meiotic marker) that developed in the cultures, but showed a significant effect on the percentages of the developed ACROSIN-positive cells (meiotic/postmeiotic marker), mainly at the higher concentration of PEDF (1000 ng/mL). On the other hand, the expression levels of ACROSIN were similar to CT. These results may suggest a possible role of PEDF in the regulation of spermatogonial cells development at the late stage of meiosis and/or postmeiotic stages. The effect of PDEF could be directly on the spermatogonial cells or developed spermatogenic cells by autocrine/paracrine effects. This effect could be also as a result of induction of the activity of the somatic cells that express PEDF-R and also produce PEDF, and their products may affect the spermatogenic process. The presence of PEDF-R in spermatozoa inside the seminiferous tubule may suggest effect of PEDF on the functionality of the developed spermatozoa.

Thus, this is the first study that shows the presence of PEDF in spermatogenic cells and testicular somatic cells, and the presence of PEDF receptor in cells of the testicular interstitial compartment and seminiferous tubules. Therefore, we suggest considering PEDF as a testicular autocrine/paracrine factor that may be involved in the regulation of testicular development and functions, including spermatogenesis.

This study may deepen our understanding of the physiological testicular autocrine/paracrine factors involved in the process of normal spermatogenesis.

## 4. Materials and Methods

### 4.1. Animals

This study was performed in accordance with the Guiding Principles for the Care and Use of Research Animals Promulgated by the Society for the Study of Reproduction. The animal protocols used in this work were evaluated and approved by the Animal Use and Ethic Committee (CEUA) of the Institute Pasteur Montevideo (Protocol 2009_1_3284, permission code, 6 August 2015). The study was confirmed by the Ben-Gurion University Ethics Committee for Animal Use in Research (IL-17-11-2014). Sexually immature and mature ICR (CD-1) (Institute of Cancer Research) male mice (Envigo Laboratories, Jerusalem, Israel) were used.

### 4.2. Preparation of Testicular Homogenates

The tunica albuginea of the testes was removed. Its content was stored in a protease inhibitor solution (1:250; Sigma, St. Louis, MO, USA) and was homogenized (with the tissues in ice). Lysates were centrifuged (13,000 rpm for 15 min at 4 °C), and the upper phase was filtered through a 0.45 µm filter (Merck KGaA, Darmstadt, Germany) and stored at −80 °C.

### 4.3. Isolation of Tubular/Spermatogonial Cells

The tubular cells were isolated from the testes of healthy, 7-day-old mice. The testicular cell suspensions were obtained as mentioned by Elhija et al., 2012 [21]. Shortly, tunica albuginea was removed and seminiferous tubules were mechanically digested and immersed in sterile phosphate-buffered saline (PBS) (Biological Industries, Beit HaEemek, Israel). After centrifugation testes were digested enzymatically (collagenase type V and DNAse) for 15 min at 37 °C. The cell suspension was filtered through a sterile cell strainer (70 µm; BD Biosciences) and washed with PBS. After centrifugation, the pellet of the cells was suspended in 1 mL of fresh StemPro-34 with 10% knock-out serum replacement (KSR) (Thermo Fisher Scientic, Waltham, MA, USA) and were counted under phase-contrast microscopy in hemocytometer.

### 4.4. Preparation of Conditioned Media from Sertoli Cell Cultures

Sertoli cell cultures were prepared from seminiferous tubules of mice from different ages (1, 4, 8 and 12-week-old) as described by Huleihel et al., 2013 [22]. Conditioned media was collected 24 h after the last overnight incubation.

### 4.5. Culture of Isolated Spermatogonial Cells in Methylcellulose Culture System (MCS)

Isolated cells from previous procedure were cultured 2 × 10^5^ cells/well/500 µL) in methylcellulose (R&D systems, Minneapolis, MN, USA) (42%), as a three-dimensional (3D) culture system including 38% StemPro-34 medium and 10% knock-out serum replacement (KSR) (Thermo Fisher Scientic, Waltham, MA, USA) and growth factors (rEGF, rGDNF, rLIF, r-bFGF) as described at AbuMadighem et al., 2018 [23]. Additionally, PEDF (Cloud-clone corp, Katy, TX, USA) in the following concentrations was added to the cultures: 1000 ng, 100 ng, 10 ng, 1 ng (Control wells did not include PEDF at any concentration). The cells were incubated for 4–5 weeks in a CO_2_ incubator at 37 °C. Every 7–10 days, 50 µL/well of fresh concentrated (×10) enriched StemPro-34 medium (containing growth factors used in the primary culture) were added to the cell cultures. At the end of the incubation period, 0.5 mL of PBS was used to suspend the cultured cells. The suspension was collected, centrifuged and re-suspended in small volume, then smeared on a slide for histological examination and/or collected and kept at −70 °C in a lysis solution for RNA extraction.

### 4.6. Testicular Tissues and Cells Immunostaining

Tissue immunostaining was performed as described previously [21,23,24,25]. Fixation of testicular tissues was done in Bouin’s solution (Kaltek, Italy), then tissues were paraffin-embedded and 5 µm section of tissues were placed on superfrost plus slides (Thermo, Braunschweig, Germany). Primary antibodies used for tissues staining in this study: rabbit polyclonal to PEDF (bs-0731R, Bioss, 1:200), goat polyclonal to α-SMA (ab21027, Abcam, 1:200), goat polyclonal to vimentin (sc-30820, Santa cruz, 1:50), goat polyclonal to 3β-HSD (sc-7557, Santa cruz, 1:100), mouse monoclonal to PEDF-R (NBP1- 25852AF647, Novus Biologicals, 1:500). Secondary antibodies used in this study: donkey anti-rabbit IgG (Cy3) (Jackson immunoresearch, 1:1000), donkey anti-goat IgG (Alexa Fluor 488) (Jackson immunoresearch, 1:100). DAPI was used for nuclei staining. Negative control tissues were incubated in a blocking buffer instead of first antibody. The stained slides were examined using a fluorescence microscope (Nikon Eclipse 50 I; Tokyo, Japan).

Tissues immunostaining was performed as described previously [21,23,24,25]. Cells were fixed in cold methanol for 20 min. The process performed for the immunostaining of testicular cells was similar to immunostaining of testicular tissues, except for antigen retrieval. Primary antibodies used for cells staining in this study: rabbit polyclonal to PEDF (bs-0731R, Bioss, 1:200), goat polyclonal to α-SMA (ab21027, Abcam, 1:200), goat polyclonal to vimentin (sc-30820, Santa cruz, 1:50), goat polyclonal to 3β-HSD (sc-7557, Santa cruz, 1:100), goat polyclonal to Integrin α-6 (sc-6596, Santa cruz, 1:30), goat polyclonal to GFR-α (sc-6157, Santa cruz, 1:50), goat polyclonal to CDH1 (E-Cadherin) (AF748,R&D Systems, 1:100), mouse monoclonal to PEDF (orb-372324, Biorbyt, 1:200), Rabbit polyclonal to CREM (12131-1-AP, Proteintech, 1:200), goat polyclonal to BOULE, Rabbit polyclonal to Acrosin (ab203289, Abcam, 1:2000). Rabbit polyclonal to VASA (NBP2-24558, Novus Biologicals, 1:50). Secondary antibodies used in this study: donkey anti-rabbit IgG (Cy3) (Jackson immunoresearch, 1:1000), donkey anti-goat IgG (Alexa Fluor 488) (Jackson immunoresearch, 1:100), goat anti-mouse IgG (Cy3) (Jackson immunoresearch, 1:500), goat anti-rabbit IgG (Alexa Fluor 488) (Jackson immunoresearch, 1:100). The stained slides were examined using a fluorescence microscope (Nikon Eclipse 50 I; Tokyo, Japan).

### 4.7. Preparation of Testicular Homogenates and Total Protein Quantification

Testicular homogenates prepared from 1, 4, 8, 12-week-old mice were performed as described at Huleihel et al., 2013 [22]. PEDF protein levels in testicular homogenates and Sertoli cell conditioned media were examined by ELISA (Cloud-clone corp., TX, USA). All reagents were prepared according to kit instruction.

### 4.8. Gene Expression–PCR Amplification

#### Real-Time Quantitative PCR

Enzymatically isolated testicular cells and cells developed in cultures were mixed with lysis buffer and 2-mercaptoethanol mixture (10 µL 2-ME/1 mL lysis solution) (GenElute Total RNA Miniprep Kit; Sigma, St. Louis, MO, USA). Synthesis of cDNA was performed according to the qScript cDNA Synthesis Kit (Quantabio, Beverly, MA 01915, USA), using random hexamers, and qPCR was performed using specific primers for PEDF, PEDF-R and spermatogenesis markers as presented in Table 1.

The reactions were conducted following the 2 × qPCRBIO SyGreen Blue Mix Hi-ROX (PCR Biosystems Ltd., Aztec House, 397–405 Archway Road, London, UK) protocol and were performed using the LightCycler 96 real-time PCR machine (Roche, Roche Diagnostics Corporation, Roche CustomBiotech, Indianapolis, IN, USA). The PCR profile included a three-step amplification program and subsequent melting, described as follows: pre-incubation (1 cycle) 95 °C for 120 s; amplification (45 cycles) 95 °C for 10 s, 60 °C for 10 s, 72 °C for 10 s; melting (1 cycle) 95 °C for 10 s, 65 °C for 60 s, 97 °C for 1 s. The PCR products were identified and distinguished using the melting curve excepted. The relative quantity of the gene expression was analyzed using the 2−∆∆*C*_t_ method. The results were expressed as the fold of increase related to the GAPDH of the same examined sample.

### 4.9. Data Handling and Statistical Evaluation

The statistical analysis for cells immunofluorescence staining results was performed by Chi-squared test with the program: The EpiInfo version 6—Statcalc.

The statistical analysis for qPCR and ELISA results was performed by t-tests with the program PRISM.

## Figures and Tables

**Table 1:** Primers used to evaluate the expression levels of factors or spermatogenic markers.

Factor/Marker		Primers
PEDF	forward	AGGCGAACTTACCAAGTCTCTG
	reverse	TGTTCCACTTGGGTGAGCTT
PEDF-R	forward	TCAGGCGAGAGTGACATCTG
	reverse	GTTGGGTTGGTTCAGTAGGC
VASA	forward	AGTATTCATGGTGATCGGGAGCAG
	reverse	GCAACAAGAACTGGGCACTTTCCA
BOULE	forward	AACCCAACAAGTGGCCCAAGATAC
	reverse	CTTTGGACACTCCAGCTCTGTCAT
ACROSINE	forward	TGTCCGTGGTTGCCAAGGATAACA
	reverse	AATCCGGGTACCTGCTTGTGAGTT
GAPDH	forward	ACCACAGTCCATGCCATCAC
	reverse	CACCACCCTGTTGCTGTAGCC

## Data Availability

The data presented in this study are available on request from the corresponding author.
